# Potential of natural stimulants and spirulina algae extracts on Cape gooseberry plant: A study on functional properties and enzymatic activity

**DOI:** 10.1002/fsn3.4342

**Published:** 2024-09-20

**Authors:** Rasoul Heydarnajad Giglou, Mousa Torabi Giglou, Mehrnaz Hatami, Mansour Ghorbanpour

**Affiliations:** ^1^ Department of Horticultural Sciences, Faculty of Agriculture and Natural Resources University of Mohaghegh Ardabili Ardabil Iran; ^2^ Department of Medicinal Plants, Faculty of Agriculture and Natural Resources Arak University Arak Iran

**Keywords:** algae extract, enzymatic activity, natural growth stimulants, taste index, total antioxidant content, total carbohydrate content

## Abstract

The need to discover new strategy to enhance agricultural productivity while minimizing negative effects on cultivation system is the driving force behind much novel research. Little is known about the use of natural growth promoters, in combination with Spirulina algae extract (*Sae*), on plants' quality and quantity characteristics. This can be an effective strategy to reduce the impact of environmental stress on plants. The aim of this study was to investigate the effects of natural growth promoters in combination with *Sae* on the functional properties and enzymatic activity of Cape gooseberry plants. For this purpose, natural growth stimulants, Kitoplus® and Azotobacter, were foliar applied to plants in a completely randomized factorial design under greenhouse conditions, with varying concentrations of *Sae*. Results indicated that the use of *Sae* in conjunction with natural growth promoters had a significant impact on increasing the yield of Cape gooseberry plants. The study found that using Kitoplus® and Spirulina extract at a concentration of 0.5% increased the yield of Cape gooseberry plants by 35.82% compared to control plants, with a yield of 310.43 g per plant. The leaves of *Physalis* plants treated with 0.5% Kitoplus®, Azotobacter biofertilizer, and *Sae* had the highest amount of chlorophyll, which was observed to be 46.2%, 40.1%, and 32.3% higher than that of control, respectively. Exposure to Kitoplus® (0.5%) and Azotobacter biofertilizer (0.5%) resulted in the highest percentage (25.49%) of dry to fresh weight ratio (DF) per fruit in the plant. Additionally, plants treated with Kitoplus® at 0.5% in combination with Azotobacter (0.5%) and *Sae* (0.5%) showed a 14.83% increase in vitamin C (VITC) levels compared to control plants. The highest levels of total antioxidant content (73.8%) and total carbohydrate content were obtained from plants treated with Spirulina algae extract spray at a concentration of 0.5% in the presence of Kitoplus® and Azotobacter. Results also showed that the highest activity of peroxidase (POD) (by 43.5% and 38.8%) and ascorbate peroxidase (APX) (by 29.1% and 28.4%) enzymes was observed in plants sprayed with natural growth promoters in the presence of Spirulina algae extract at concentrations of 0.25% and 0.5%, respectively. Overall, this research opens the possibility of utilizing foliar spray of effective biostimulants in combination with *Sae* to improve the quantity and quality of Cape gooseberry plants. Implementing this simple, safe, eco‐friendly, and low‐cost management tool can result in nutritionally rich fruit that benefits the consumer at little or no additional cost.

## Graphical abstract

Natural growth stimulants (Kitoplus® and Azotobacter at concentrations of 0% and 0.5%, combined with Spirulina algae extract at concentrations of 0%, 0.25%, and 0.5%) were applied to Cape gooseberry plants.

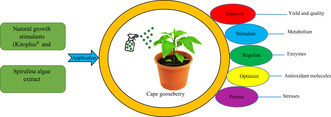

## 1 INTRODUCTION

Fruits and vegetables are essential for maintaining good health as they provide the body with necessary nutrients. They help in reducing the risks of many diseases, including cardiovascular disease and certain types of cancer (Arts & Hollman, 2005; Leenders et al., 2014). The health benefits of fruits and vegetables are due to the presence of vitamins, minerals, dietary fibers, and a wide range of chemical and phytochemical substances. These substances help in maintaining the proper levels of nonenzymatic and enzymatic antioxidant defenses and protect the body from free radicals (Abdel Moneim, 2014).

*Physalis peruviana* L., also known as Cape gooseberry, is a fruit from the *Solanaceae* family that contains health‐promoting compounds (Martinez, 1998). Since the 1980s, Cape gooseberry has been commercially produced and is now available in many countries across Europe, North America, Asia, and Oceania. However, the production and nutritional value of this fruit are limited by specific challenges. For example, Cape gooseberry is highly perishable, and its quality is threatened by a variety of factors during cultivation and after harvest (Heydarnajad Giglou & Torabi Giglou, 2023). This inefficiency is caused by issues, such as farmers' poor flexibility and limited knowledge of consumer priorities, as highlighted by Wang (2014).

There have been several recent studies exploring how a mother plant's environment, including factors such as temperature, light, and nutrition, can affect the quality of its fruit and seedlings (Bakhoum et al., 2022; Sadak et al., 2016). However, there is still a lack of information about how a plant's nutritional conditions can optimize its yield and impact the biochemical characteristics of its fruit. Additionally, a plant's performance is a complex feature that is influenced by environmental conditions, cultivation systems, and nutritional fertilizers. Therefore, it is crucial to prioritize the management and application of more efficient fertilizers that are both economically and performancewise beneficial. Microalgae are important biological resources that have a balanced chemical composition and can be used to produce various novel products and applications. They can be utilized to enhance the nutritional value of food and animal feed, and to produce organic fertilizers. Microalgae contain valuable substances, such as unsaturated fatty acids, pigments, antioxidants, medicinal compounds, and other bioactive compounds. According to a study conducted by Bakhoum et al. (2023), the combined treatments of foliar spray with 1.5 g L^−1^ of algae extract are highly recommended for improving wheat growth and productivity under reduced nitrogen fertilizer rates and water deficit.

*Spirulina platensis*, also known as Spirulina, is a type of microalgae that is considered a “food‐medicine” due to its rich and varied secondary metabolites, bioactive pigments like carotenoids and phycobilins, and well‐balanced chemical composition (Padash et al., 2023). Belay (2008) determined the chemical composition of Spirulina, introducing it as a nutritional supplement with high nutritional value. It contains 16.6% carbohydrates, 6.3% fat, 44.9% protein, and 8.9% ash or minerals. The Food and Drug Administration (FDA) has recognized Spirulina microalgae supplements as “generally recognized as safe,” making it a dependable source for food products and a natural growth stimulant (Foo et al., 2017; Lucas et al., 2018). Microalgae biostimulants are used in combination with inorganic fertilizers to improve crop growth and yield (Miranda et al., 2024; Parmar et al., 2023). These biostimulants are used in small quantities and have been found to enhance chlorophyll content, antioxidants, metabolism, and the shelf life of harvested products (Rady et al., 2023; Solomon et al., 2023). Bioactive substances present in Spirulina liquid extracts have great potential to be used as biostimulants for plant growth in agriculture (Abreu et al., 2023). Previous studies have also shown that *Spirulina*‐based biostimulants can improve the mineral status of soil and have a positive effect on the yield of plants (Godlewska et al., 2019).

It is important to modify fertilization methods to ensure that plants have access to the necessary nutrients over a long period without any losses. This is because the use of chemical fertilizers at the beginning of the agricultural season may cause plants to use them up after converting them into other forms or be washed away by irrigation water. One of the best agricultural practices to address this issue is the use of nitrogen‐fixing biofertilizers. According to a study by Nosheen et al. (2021), this method can significantly increase nutrient use efficiency. Furthermore, other studies have shown that growth‐promoting bacteria can improve plant growth by enhancing nitrogen uptake, reducing the amount of chemical fertilizers used, and increasing their efficiency (Kumar et al., 2022). Biological fertilizers are made up of living cells of various microorganisms that can absorb nutrients through biological processes and provide them to plants. According to studies conducted by Karimzadeh Asl et al. (2018) and Gu et al. (2023), these fertilizers have been found to be effective. A study by Azzaz et al. (2009) explored the possibility of using biological fertilizers instead of chemical fertilizers in fennel plants. The results showed that fennel plants treated with biological fertilizers had better vegetative growth, higher yield, and increased essential oil production.

Kitoplus® is a product that contains chitin compounds. These compounds are the main components of the cell walls of some animals, including crustaceans like shrimp and crabs, insects, some plant pathogens, and microorganisms. Kitoplus® has various applications in industries, such as pharmaceuticals, agriculture, and others (Torabi Giglou et al., 2020). Studies have shown that chitosan and its derivatives can act as effective biostimulants that increase the production of secondary metabolites in plants (Cheng et al., 2006; Sadak et al., 2022). Chitosan has also been found to increase drought tolerance in plants by reducing transpiration and maintaining relative water content (Mohammadi et al., 2021). In a study investigating the stimulatory effect of chitosan polymers on eggplant plants, it was found that chitosan stimulants can increase the activity of plant enzymes, such as chitosanase and peroxidase (POD) (Mandal, 2010).

There have been several applications of biochemical fertilizers and seaweed extracts in agriculture, food industries, and animal sciences. One such new application is the use of Spirulina algae extract, which contains protein and amino acids, as a combination of nutrients to provide necessary elements for plants. However, there have been limited studies conducted on the effectiveness of Spirulina algae extract in agriculture. Therefore, it is necessary to conduct more research in this area, given the economic importance and increasing demand for natural biostimulants and elicitors. This study aims to investigate the effects of Spirulina algae extract (*Sae*) and plant growth stimulants (Kitoplus®) on the growth of *Physalis* plants.

## 2 MATERIALS AND METHODS

### 2.1 Algae cultivation and extraction

For the cultivation of Spirulina algae, Zarrouk's medium (Zarrouk) was used according to our previous study method (Padash et al., 2023). Then, the cultured samples were harvested and dried at 45°C for 72 h. For the extraction of the extract from the algae, the dried samples were first soaked in 80% methanol for 72 h. The samples were sonicated three times for 15 min. Then, the obtained extracts were used after filtration. The results of the extraction method are published in our previous study (Padash et al., 2023).

### 2.2 Plant growth and treatments

The *Physalis peruviana* seeds (Accession no: TN‐82‐765) used in this study were provided by The National Plant Gene‐Bank of Iran. Then, Cape gooseberry seeds were cultured in petri dishes and transferred to cultivation trays after germination. The seedlings were kept in the cultivation trays for 30 days after the appearance and growth of the stems. Then, the plants were transferred to the greenhouse, and after proper acclimatization (2 weeks later) in pots, the treatments were applied. The present experiment was conducted in a completely randomized factorial design with four replicates, including *Sae* spray solution at three concentrations (0%, 0.25%, and 0.5%), Azotobacter biofertilizer (produced by Alborz Rahvar Biotechnology, Iran) at two concentrations (0% and 0.5%), and Kitoplus® growth stimulator (produced by Kimia Sabz Avar, Iran) at two concentrations (0% and 0.5%), based on the results of our previous studies (Giglou et al., 2022; Torabi Giglou et al., 2020), with eight Cape gooseberry plants in each replicate. The spray solution was applied in three stages, every 15 days. One week after the final spray, traits, such as fresh and dry fruit weight, plant yield, titratable acidity, total soluble solids, taste index, and other biochemical traits along with antioxidant enzyme activity, were measured.

### 2.3 Total soluble solids and titratable acidity

The total soluble solids (TSS) were measured at room temperature (25 ± 2°C) using a refractometer (K‐0032 manufactured in Japan). The amount of TSS was recorded in degrees Brix. The titrable acidity (TA) was determined by titrating 1 mL of diluted extract in 25 mL of distilled water to pH 8.2, using 0.1 N sodium hydroxide (NaOH) as per Ayala‐Zavala et al. (2004). The taste index (TSS/TA) was obtained by dividing the TSS by the total value of TA.

### 2.4 Calculation of photosynthetic pigments

To determine the levels of plastid pigments, chlorophylls, and carotenoids, in fresh fruit pericarp, we used Arnon's method (1967). We took a 1 g sample of the pericarp and extracted chlorophyll and carotenoids using acetone of 80% concentration (v/v). We measured the absorption at 645 and 663 nm for chlorophyll, and 480 and 510 nm for carotenoids, using a spectrophotometer (UV‐600) from HACH Company, Loveland, USA. The concentration of total chlorophyll and carotenoids was expressed as 100 mg per g of fresh weight (FW) and was calculated using the following formula:

$$\text{Total chlorophylls}=\left[20.2\;\left(A_{645}\right)+8.02\;\left(A_{663}\right)\right]\times V/W\;1000;$$

$$\text{Total carotenoids}=\left[7.6\left(A_{480}\right)–1.49\left(A_{510}\right)\right]\times V/W1000.$$

### 2.5 Vitamin C

In order to determine the amount of vitamin C (VITC) contained in a berry extract, a titration method was employed using iodine in potassium iodide (KI). The end of the titration was identified by observing a consistent dark blue color of the berry extract for several seconds (Arya, 2000).

### 2.6 Preparation of berries powder for measuring phenol, flavonoids, and antioxidant contents

The process involved in the preparation of the sample was to dry the fruits in an oven at a temperature of 40°C for 96 h. After this, the dried material was milled. Then, 1 g of each sample was soaked in 50 mL of 80% methanol for 48 hours at room temperature. Once the 48‐h period was over, the extracts were filtered using Whatman No. 4 filter paper. The solvent was then evaporated at a temperature lower than 55°C. All further experiments were carried out at 4°C, following the procedure described by Pourmorad et al. (2006).

### 2.7 Total phenol content

To measure the total phenol content (TPC) of a plant extract, mix 2 mL of 2% sodium carbonate (Na_2_CO_3_), 3 mL of distilled water, and 100 μL of Folin–Ciocalteu's phenol reagent (50%) with 100 μL of the extract. Record the absorbance for half of the reaction time at a wavelength of 720 nm compared to a control. Draw a standard curve using gallic acid as the standard. Calculate and report the total phenol content of the plant extract per milligram (mg) (equivalent to glycogen acid per gram of dry weight of the plant) using the standard curve. This method was developed by Meda et al., 2005.

### 2.8 Total antioxidant capacity

To evaluate the antioxidant activity of the extract, we used the 2,2‐diphenyl‐1‐picrylhydrazyl (DPPH) method, as described by Miliauskas et al. (2004). We mixed various concentrations of the extract with 2 mL of a methanolic solution containing 0.004% DPPH. The final mass ratio of the extract to DPPH was approximately 3:1, 1.5:1, and 0.75:1, respectively. We also prepared a control solution containing 2 mL of DPPH and 2 mL of methanol. The solutions were then kept in the dark at room temperature for 30 min. After this, we measured the absorbance of the samples at 517 nm against the methanol control. Finally, we calculated the percentage of free radicals (I%) in each extract using the following formula:

$$\%I=(A_{\text{CONTROL}}–A_{\text{SAMPLE}})/A_{\text{CONTROL}}\times 100.$$

### 2.9 Estimation of catalase and ascorbate peroxidase activity

To determine the antioxidant enzymes, a sample of 0.1 g of fresh leaves was taken and mixed with 1.5 mL of phosphate‐buffered saline (PBS) (pH: 7.4) containing 1 g of polyvinylpyrrolidone (PVP), 0.8 g of potassium chloride (KCl), 0.8 g of sodium chloride (NaCl), 0.14 g of sodium hypophosphite (Na_2_HPO_2_), and 0.02 g of potassium dihydrogen phosphate (KH_2_PO_4_) on ice. The mixture was then homogenized using a Chinese mortar and centrifuged at 10,000 rpm (revolutions per minute) for 10 min at 45°C. The resulting supernatant was then used to assay the catalase (CAT) and ascorbate peroxidase (APX) activities.

To measure the CAT activity in leaves, the method developed by Boominathan and Doran (2002) was used. To do this, 900 μL of a reaction solution consisting of a 10 mM hydrogen peroxide (H_2_O_2_) solution in phosphate‐buffered saline without PVP, along with 100 μL of enzyme extract in a glass cell, was prepared. After adding H_2_O_2_ to the reaction solution, the decrease in its concentration was immediately measured at a wavelength of 240 nm using a spectrophotometer within 1 min. The amount of CAT activity was then calculated based on these measurements.

The measurement of APX activity was carried out using the method developed by Boominathan and Doran (2002). To initiate the reaction, 900 μL of the reaction solution was used, which contained 625 μL of phosphate buffer containing ethylenediaminetetraacetic acid (EDTA), 175 μL of ascorbic acid, 50 μL of H_2_O_2_, 50 μL of bovine serum albumin (BSA), and 100 μL of enzyme extract. The reaction mixture was then poured into a glass cell, and the spectrophotometer was used to measure the reduction of ascorbic acid due to the activity of the corresponding enzyme at a wavelength of 290 nm for one minute. Finally, the value of APX activity was determined.

### 2.10 Estimation of peroxidase activity

To measure the activity of the peroxidase (POD) enzyme, we followed the method described by Upadhyaya et al. (1985). We prepared a reaction mixture by combining 2.5 mL of 50 mM phosphate buffer (pH = 7), 1 mL of 1% guaiacol, 1 mL of 1% hydrogen peroxide, and 0.1 mL of extract. The peroxidase enzyme activity was then calculated by measuring the increase in absorbance at a wavelength of 420 nm for 1 min using a spectrophotometer. The activity level of extinction coefficient (26.6 mM^−1^ cm^−1^) was measured using the following equation.

$$\text{Units}\left(\mathrm{Mm}/\min\right)=\frac{{\frac{\mathrm{doD}}{\min\left(\text{slop}\right)}}^{*}\mathrm{Vol}.\text{of assay}\left(0.0003\right)}{\text{Extinction cofficient}\;\left(0.0436\right)}$$

### 2.11 Determination of total carbohydrate content (TC)

To determine the total carbohydrates (TC) present in a sample of dry plant tissue, the phenol–sulfuric acid method was used. First, 0.1 g of the tissue was added to 10 mL of 70% ethanol and kept at 4°C for 1 week, with daily stirring. This allowed the dissolved carbohydrates to separate from the rest of the tissue. After a week, 1 mL of the supernatant solution was taken from each sample and diluted with distilled water to make it up to 2 mL. Then, 1 mL of 5% phenol and 5 mL of 48% sulfuric acid (H2SO4) were added to the samples and mixed well by vortexing. The test tubes were then placed in a hot water bath at 65°C for 20 min and then cooled to room temperature to stop the reactions. The amount of absorption was measured using a spectrophotometer at a wavelength of 485 nm. A standard curve was prepared using solutions with concentrations ranging from 0 to 10 mg/100 mL of glucose. To calculate the amount of TC in the samples, the mg g^−1^ of dry weight of the samples was determined (Irigoyen et al., 1992), taking into account the dry weight of the samples. Statistical analyses were performed to analyze the data obtained from the measurements.

### 2.12 Statistical analyses

The data were analyzed statistically using the SPSS software package v. 20.0 for Windows by SPSS (SPSS Inc., Chicago, IL, USA). Duncan's multiple range tests were used to compare the means at a significance level of *p* < .05. If there were differences significant at *p* < .05, they were indicated using different letters. The data are shown as the mean ± SD.

## 3 RESULTS

### 3.1 Yield‐related traits

**FIGURE 1:**
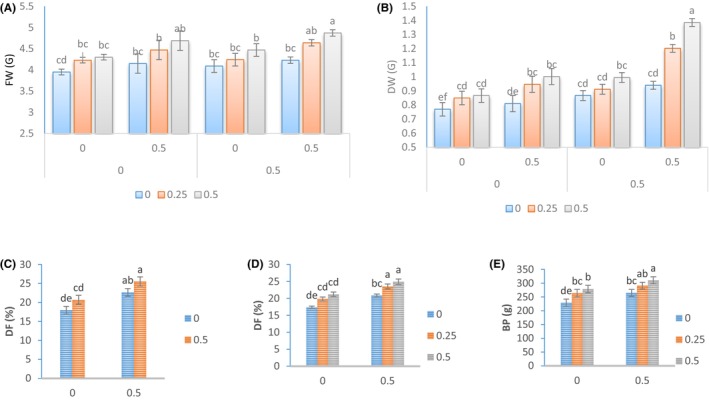
Changes in the yield traits of Cape gooseberry fruits under the application of *Sae* and natural growth stimulants. The *Sae* included concentrations of 0%, 0.25%, and 0.5%, and the natural growth stimulants Kitoplus® and Azotobacter biofertilizer included concentrations of 0% and 0.5%.

In order to study fruit yield traits, we measured the fresh weight (FW), dry weight (DW), dry to fresh weight ratio (DF), and plant yield (BP) of plants that were subjected to different treatments. Our findings indicate that the application of Azotobacter biofertilizer, Kitoplus® growth stimulator, and *Sae* together had a significant impact on the yield traits. The plants treated with a spray solution of Kitoplus® at 0.5% concentration, Azotobacter biofertilizer at 0.5% concentration, and *Sae* at 0.5% concentration showed the highest fresh weight (FW) (Figure 1A) and DW (Figure 1B) values, with 4.87 and 1.385 g per fruit, respectively. In the study, it was found that the treatment of Kitoplus® at 0.5% concentration and Azotobacter biofertilizer at 0.5% concentration resulted in the highest percentage of DF per fruit in the plant (25.49%) (Figure 1C). Similarly, the treatment of Azotobacter biofertilizer at 0.5% concentration and *Sae* at 0.5% concentration resulted in 24.89% DF per fruit in the plant (Figure 1D). When it comes to the effect of these treatments on BP, it was observed that the plants treated with the spray solution of Kitoplus® and *Sae* at 0.5% concentration had the highest BP, which was 36.46% higher than that of the control plants (Figure 1E).

### 3.2 Chlorophyll and carotenoids

**FIGURE 2:**
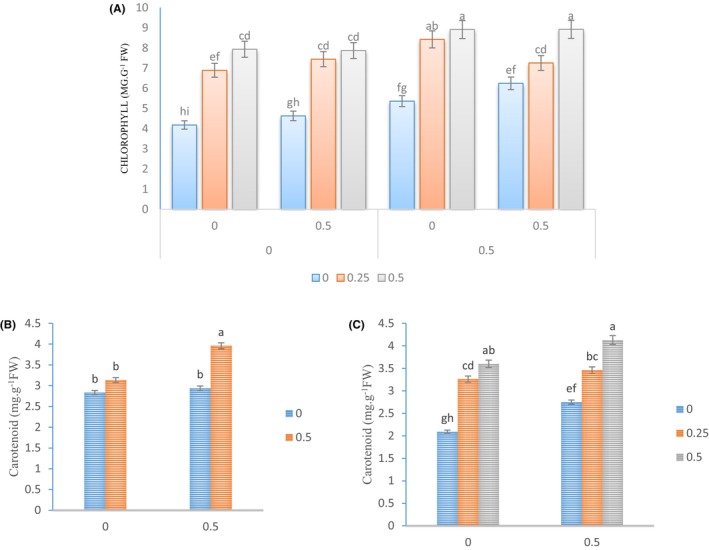
Changes in the photosynthetic pigments in the leaves of Cape gooseberry plants under the application of Spirulina algae extract and natural growth stimulants. The *Sae* included concentrations of 0%, 0.25%, and 0.5%, and the natural growth stimulants Kitoplus® and Azotobacter biofertilizer included concentrations of 0% and 0.5%.

The levels of chlorophyll and carotenoid in all plants that were treated with Kitoplus® and Azotobacter biofertilizer increased as the concentration of *Sae* spray increased. The leaves of *Physalis* plants treated with Kitoplus® (at 0.5% concentration), Azotobacter biofertilizer (at 0.5% concentration), and *Sae* at 0.5% concentration had the highest amount of chlorophyll, which was observed to be 46.2%, 40.1%, and 32.3% higher than that of control, respectively (Figure 2A). Moreover, the spray solution of Kitoplus® and Azotobacter biofertilizer at 0.5% concentration had a positive effect on carotenoid levels. The highest level of carotenoid was observed in the plants treated with this spray solution (Figure 2B). Our results also showed that the treatment of *Sae* at 0.5% concentration in the presence of Kitoplus® at 0.5% concentration had the highest carotenoid level in the leaves of plants (Figure 2C).

### 3.3 Vitamin C, TSS, TA, TSS/TA ratio, and TC

**FIGURE 3:**
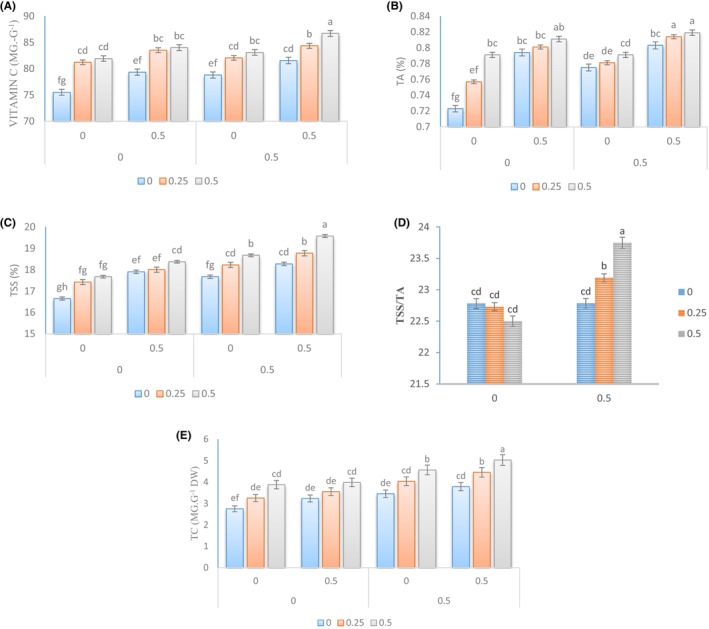
Changes in the TSS (A), TA (B), vitamin C (C), TSS/TA (D), and TC (E) in the leaves of Cape gooseberry plants under the application of *Sae* and natural growth stimulants. The *Sae* included concentrations of 0%, 0.25%, and 0.5%, and the natural growth stimulants Kitoplus® and Azotobacter biofertilizer included concentrations of 0% and 0.5%.

The concentration of Spirulina algae extract affected the levels of vitamin C, TSS, total antioxidant (TA) chlorophyll, TSS/TA ratio, and total carbohydrates (TC) in plants, with or without Kitoplus® and Azotobacter biofertilizer (Figure 3A–E). The plants treated with Spirulina algae extract at a concentration of 0.5% had the highest levels of vitamin C (70.80 mg g^−1^), TA (0.819%), and TSS (19.57%). The taste index (TSS/TA) was also highest in plants treated with Spirulina algae extract and Kitoplus®. Additionally, the level of TC increased with the presence of Kitoplus® and Azotobacter biofertilizer when using Spirulina algae extract, with the highest level of TC (0.235 g.100 g^−1^ dry weight) obtained at a concentration of 0.5%.

### 3.4 TPC and TAO levels

**FIGURE 4:**
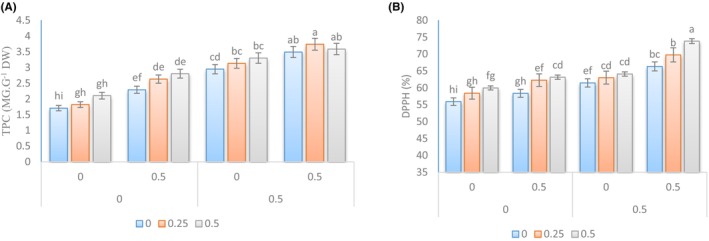
Changes in TPC (A) and DPPH (B) in the leaves of Cape gooseberry plants under the application of *Sae* and natural growth stimulants. The *Sae* included concentrations of 0%, 0.25%, and 0.5%, and the natural growth stimulants Kitoplus® and Azotobacter biofertilizer included concentrations of 0% and 0.5%.

In this study, the effects of different treatments on the levels of total phenol content (TPC), total antioxidant (TAO), vitamin C, and total carbohydrates were analyzed (Figure 4A,B). The results showed that the use of *Sae* led to an increase in the levels of TPC and TAO. The highest level of TPC (74.3 mg g^−1^ DW) was observed when *Sae* was used at a concentration of 0.25% along with Kitoplus® and Azotobacter biofertilizer. This level was not significantly different from the level obtained when *Sae* was used at a concentration of 0.5%. Similarly, the highest level of total antioxidant (8.73%) was observed when *Sae* was applied at a concentration of 0.5% along with Kitoplus® and Azotobacter biofertilizer. These results suggest that the use of *Sae* along with Kitoplus® and Azotobacter biofertilizer can enhance the levels of TPC and TAO in plants.

### 3.5 Protein and activity of antioxidant enzymes

**FIGURE 5:**
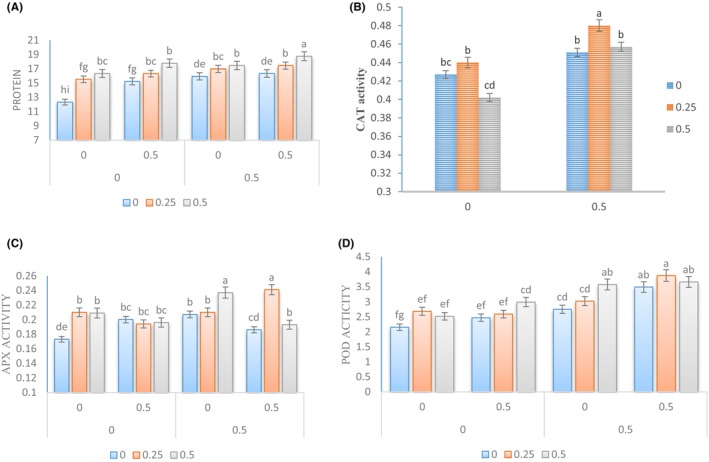
Changes in the protein and antioxidant enzymes in the leaves of Cape gooseberry plants under the application of *Sae* and natural growth stimulants. The *Sae* included concentrations of 0%, 0.25%, and 0.5%, and the natural growth stimulants Kitoplus® and Azotobacter biofertilizer included concentrations of 0% and 0.5%.

The research conducted on plants treated with natural growth stimulants and *Sae* showed a significant increase in protein levels. This increase had a noticeable effect on the activity of antioxidant enzymes. Our findings indicated that the highest protein level was obtained when plants were sprayed with *Sae* at a concentration of 0.5% and under the conditions of simultaneous use of Kitoplus® and Azotobacter biofertilizer (Figure 5A). Furthermore, the examination of the activity level of CAT enzyme also showed a rise in the treatment of foliar application of *Sae*. The highest activity level of this enzyme was observed under the conditions of using *Sae* at a concentration of 0.5% and Kitoplus® natural growth stimulant (at a concentration of 0.5%) (Figure 5B).

During an examination of enzyme activity levels, it was observed that the APX activity decreased with an increase in *Sae* concentration when used in conjunction with Azotobacter biofertilizer. However, when foliar application of *Sae* was used in conjunction with both natural growth stimulants Kitoplus® and Azotobacter, the APX activity level increased noticeably. The highest activity level of the APX was observed when using Kitoplus® and Azotobacter natural growth stimulants at a concentration of 0.5% each, along with a concentration of 0.5% of *Sae*. This activity level was 10.12% higher than the enzyme activity level observed in control plants, as shown in Figure 5C.

The level of activity of the POD was found to be consistent with the protein level and increased with an increase in the concentration of *Sae*. The highest level of activity of this enzyme was observed when *Sae* was used at a concentration of 0.25% in conjunction with both natural growth stimulants (Kitoplus® and Azotobacter) at a concentration of 0.5% (Figure 5D). The activity level of the POD was 42.21% higher under these conditions than in plants grown under normal conditions.

### 3.6 Analysis of the impact of different treatments on the studied indices in a Heatmap chart

**FIGURE 6:**
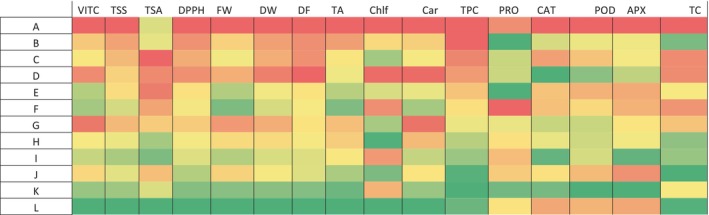
The results of Heatmap analysis in plants under the conditions of using natural growth stimulants, Kitoplus® and Aztobacter and *Sae* foliar treatment. The letters are in order (A: control, *Sae* at a concentration of 0.25%, B: *Sae* at a concentration of 0.5%, C: Azotobacter fertilizer at a concentration of 0.5%, D: Azotobacter fertilizer at a concentration of 0.5% and *Sae* at a concentration of 0.25%. 0%, E: Azotobacter at a concentration of 0.5% and *Sae* at a concentration of 0.5%, F: Kitoplus® at a concentration of 0.5%, G: Kitoplus® at a concentration of 0.5% *Sae* at a concentration of 0.25%, H: Kitoplus® at a concentration of 0.5% and *Sae* at a concentration of 0.5%, I: Kitoplus® at a concentration of 0.5% and Azotobacter at a concentration of 0.5%, J: Kitoplus® at a concentration of 0.5%, Azotobacter at a concentration of 0.5%, and *Sae* at a concentration of 0.25%, K: and Kitoplus® at a concentration of 0.5%, Azotobacter at a concentration of 0.5%, and *Sae* at a concentration of 0.5%).

The Heatmap chart is often used in studies to demonstrate the effects of treatments on different indices. In this experiment, the results of the Heatmap showed that natural growth stimulants Kitoplus® and Azotobacter, as well as *Sae*, had a positive effect. The results indicated that the most significant impact of these treatments on the levels of VITC, TSS, titratable acidity (TSA), DPPH, FW, DW, DF, TA, chlorophyll (Chlf), carotenoid (Car), total carbohydrate (TC), and TPC was achieved when using *Sae* at a concentration of 0.5% and with the simultaneous use of both natural growth stimulants Kitoplus® and Azotobacter (Figure 6).

After analyzing the activity of antioxidant enzymes, the Heatmap model revealed that the most efficient enzyme activity for APX, POD, and CAT enzymes was observed in plants that were exposed to both natural growth stimulants, Kitoplus® and Azotobacter. This was achieved through foliar application of *Sae* at a concentration of 0.25% (Figure 6).

## 4 DISCUSSION

The use of biostimulants in agriculture is expanding worldwide, particularly those derived from algae extracts. These biostimulants have properties that enhance plant growth, performance, and quality, as well as improve nutrient uptake efficiency (Arioli et al., 2020; Shukla et al., 2019). Our study found that the effect of *Sae* significantly increased various growth parameters of plants, such as plant yield, TSS, and vitamin C, which are essential indicators of *Physalis* fruit, when combined with natural growth stimulants (Kitoplus® and Azotobacter) and *Sae*. Our findings are consistent with previous reports that different concentrations of seaweed at different stages of pollination can increase grain weight, yield, and nutritional quality of plants compared to the control treatment (Colla & Rouphael, 2020; Gao et al., 2017). It has also been reported that foliar application of algae extract can enhance cytokinin synthesis, which leads to increased cytokinin availability, ultimately leading to the initiation of pollination and increased plant yield (Garcia‐Gonzalez & Sommerfeld, 2016; Paul & Yuvaraj, 2014).

Our research findings indicate that the use of *Sae* in combination with natural growth stimulants Kitoplus® and Azotobacter can increase the total carbohydrate content in *Physalis* fruits. This increase in carbohydrate content may be attributed to the rise in chlorophyll content, leading to increased photosynthesis and carbohydrate production. This aligns with the findings of Sridhar and Rengasamy (2010). Another study by Paul and Yuvaraj (2014) found that the concentration of algae extract also played a role in increasing TSS in plants. The maximum TSS and TA were observed at a 1% concentration of algae extract, consistent with our study. Furthermore, Kapoore et al. (2021) suggests that the increase in total carbohydrate content due to the application of leaf algae extract may be a result of enhanced nutrient uptake and the availability of essential elements.

According to a study by El‐Tanahy et al. (2012), the use of *Sae* appears to have increased the nitrogen fixation activity of microorganisms, leading to a subsequent increase in nitrogen fixation and protein content in *Physalis* plants. On the other hand, Kitoplus® may provide amino acid compounds necessary for plant growth, which could result in increased total nitrogen in leaves and improved ability of plants to absorb nitrogen from the soil. This is because Kitoplus, containing chitosan, can increase antioxidant enzymes, such as CAT, POD, and APX, and activate key enzymes involved in nitrogen metabolism, resulting in increased transport and transfer of nitrogen in leaves and stems.

Kitoplus, with its chitosan content, enhances the availability, absorption, and transportation of essential nutrients in plants by regulating cellular osmotic pressure. This results in improved growth and development of plants, which is evidenced by an increase in the number of leaves, branches, leaf area, and leaf surface area, ultimately leading to an increase in plant weight and dryness (Farouk & Ramadan, 2012). On the other hand, *Sae* has less auxin hormone but contains substances, such as mannitol, laminarin, gibberellin, cytokinin, and glutamic acid. When plants are fed with this algae extract, it leads to an increase in the rate of photosynthesis and the production of sugars and amino acids like Strazas (Andrea Ertani et al., 2018). It has been suggested that the increase in plant protein content may be due to the presence of phenylacetic acid or similar compounds and the presence of some growth stimulants in seaweed extract (Ertani et al., 2018; Torabi Giglou et al., 2020; Yao et al., 2020). Studies have shown that the use of this algae extract increases the chlorophyll concentration in plant leaves and increases the level of amylase enzyme in plant organs, which breaks down nonusable sugars in the plant (Yao et al., 2020).

Mallikiarjun and Jata (2019) conducted a study where they found that the application of seaweed extract spray on plants led to an increase in growth and yield compared to plants that were not treated. The results showed that as the application of the seaweed extract increased, there was a gradual increase in plant height, number of branches, number of pods, and ultimately the highest plant yield. Seaweed extracts, especially Spirulina, have several benefits, including improved plant performance, increased nutrient absorption, higher photosynthetic activity, and resistance against harmful biotic and abiotic stresses (Van Oosten et al., 2017). The rhizosphere, which is the interface between plant roots and soil, contains microbiota that are crucial for enhancing plant growth and health (Vives‐Peris et al., 2020). This is because the rhizosphere microbiota can protect plants against harmful pathogens (Mendes et al., 2011; Rolfe et al., 2019) and also aid in plant nutrition (Bulgarelli et al., 2013). The microbiota can increase the production of plant growth hormones, stimulate the plant immune system, enhance nutrient uptake, and improve soil fertility (Vives‐Peris et al., 2020). Therefore, using seaweed extracts as a natural and chemical‐free source of nutrition can significantly improve plant growth, performance, and promote the health of the rhizosphere and microbiota.

Our research has uncovered an important finding that the use of *Sae*, along with natural growth stimulants, occurs at the ecosystem level during plant growth. We believe that applying *Sae*, Kitoplus®, and Azotobacter results in better plant growth and root systems that release more exudates into the soil, leading to increased microbial biomass. This, in turn, benefits soil biogeochemical cycles. Our suggestion aligns with recent studies that indicate how plant species with nutrient acquisition traits regulate the plant–soil carbon–nitrogen feedback system that works at the root–soil interface (Vives‐Peris et al., 2020). Furthermore, it has been reported that when seaweed extract is applied to plants, there is an increase in soluble protein, antioxidant capacity, and secondary metabolites, which is accompanied by an increase in the expression of key enzymes involved in nitrogen metabolism, antioxidant capacity, and glycine betaine synthesis (Goni et al., 2018). The combined feeding method, which involves the use of natural growth stimulants along with other materials, has been observed to increase yield and enzymatic activity. This is believed to be caused by the alteration of the physical and chemical properties of the soil, leading to improved accessibility of low‐consumption elements and their absorption by plants. A study conducted by Renuka et al. (2018) suggests that Spirulina seaweed extract, when used in combination with natural growth stimulants such as chitosan and Azotobacter, may offer collective benefits for sustainable agriculture and high‐performance food production. However, further research from an agro‐ecosystem perspective is necessary to fully understand these benefits.

## 5 CONCLUSION

Based on the research findings, it can be concluded that the use of natural growth stimulants, such as Kitoplus® and Azotobacter, along with *Sae*, has a significant impact on the yield and antioxidant enzyme activities in plants. This effect is particularly noticeable in plants treated with *Sae* at a concentration of 0.5%. In general, it is possible to increase the amount of carbohydrates, vitamin C, and yield in *Physalis* plants through proper agricultural techniques. The use of biofertilizer chitosan, along with Azotobacter and *Sae*, resulted in heavier and drier fruits, and higher antioxidant enzyme activity in exposed plants compared to control. The Heatmap results showed that using *Sae* at concentrations of 25.0% and 5.0% in combination with natural growth stimulants Kitoplus® and Azotobacter (at a concentration of 0.5%) increased the levels of chlorophyll and carotenoids. This, in turn, increased the efficiency of photosynthesis and carbohydrate production. Therefore, it is recommended to use natural growth stimulants such as Kitoplus® and Azotobacter, along with *Sae* at different concentrations, instead of chemical fertilizers for achieving high yields in *Physalis*. This would support the goal of sustainable agriculture and horticulture.

## AUTHOR CONTRIBUTIONS

**Rasoul Heydarnajad Giglou:** Conceptualization (equal); data curation (equal); formal analysis (equal); investigation (equal). **Mousa Torabi Giglou:** Conceptualization (equal); formal analysis (equal); investigation (equal); methodology (equal); visualization (equal); writing – original draft (equal). **Mehrnaz Hatami:** Formal analysis (equal); resources (equal); software (equal); writing – review and editing (equal). **Mansour Ghorbanpour:** Software (equal); writing – review and editing (equal).

## CONFLICT OF INTEREST STATEMENT

The authors declare no competing interests.

## STATEMENT OF COMPLIANCE

The authors confirm that all the experimental research and field studies, including the collection of plant material, complied with relevant institutional, national, and international guidelines and legislation.

## STATEMENT ON EXPERIMENTAL RESEARCH AND FIELD STUDIES ON PLANTS

The growing plants sampled comply with relevant institutional, national, and international guidelines and domestic legislation of Iran.

## ETHICS APPROVAL AND CONSENT TO PARTICIPATE

All methods performed in this study including the collection of plant materials were in compliance with the relevant institutional, national, and international guidelines and legislation.

## Data Availability

All the data generated or analyzed during the current study were included in the manuscript. The raw data are available from the corresponding author on reasonable request.
